# Nonlinear effects of humidex on risk for asthenopia among adults: a national cross-sectional survey in China

**DOI:** 10.3389/fpubh.2025.1515672

**Published:** 2025-03-13

**Authors:** Xiaotian Wu, Xin Chen, Fuyue Tian, Maoyuan Yang, Fan Lu, Ruzhi Deng, Na Lin

**Affiliations:** National Clinical Research Center for Ocular Diseases, Eye Hospital, Wenzhou Medical University, Wenzhou, China

**Keywords:** humidex, humidity, temperature, asthenopia, risk factor

## Abstract

**Introduction:**

The health effects of ambient humidity and temperature are well-established. However, few studies have investigated the relationship between humidity, temperature, and asthenopia. Our goal was to quantify the impact of humidity and temperature on the likelihood and severity of asthenopia among adults in China.

**Methods:**

We conducted a national cross-sectional online survey from June 2020 to March 2022, involving 48,000 adults aged 18 and older from 31 provinces in China. City-level meteorological data, including daily average temperature and relative humidity, were obtained from the China Meteorological Data Network. The humidex was used as the exposure indicator. Asthenopia was self-reported using the 17-item Asthenopia Survey Questionnaire. Covariates included age, gender, season, geographic region, presbyopia status, history of eye surgery, average daily duration of near vision activities, daily sleep duration, sleep quality, and frequency of heightened anxiety or depression. The generalized estimation equation model was used to analyze the associations between humidex and asthenopia.

**Results:**

Of the 34,303 adults who completed the survey, 38.8% reported asthenopia. Among these, 17.1% were mild, 18.5% were moderate, and 3.2% were severe. The average humidex of the past 2 weeks (range − 28.25 to 45.75°C, mean 25.07 ± 14.26°C) was positively correlated with the likelihood (OR: 1.005, 95% CI: 1.003–1.006, *p*-value <0.001) and severity (OR: 1.004, 95% CI: 1.003–1.006, *p*-value <0.001) of asthenopia. The associations between the average humidex of the past 1 month or 1 year and asthenopia were consistent with the past 2 weeks. Additionally, a nonlinear J-shaped relationship was observed between humidex and asthenopia prevalence and severity: low, high, and very high humidex were identified as risk factors for asthenopia.

**Conclusion:**

Both high (≥30°C) and low (<10°C) humidex levels were associated with higher likelihoods and severity of asthenopia in adults. The influence of meteorological factors such as humidity and temperature on asthenopia should not be overlooked.

## 1 Introduction

Asthenopia, commonly known as visual fatigue or eye strain, includes a variety of subjective discomforts related to the eyes, vision, or general well-being, significantly impacting an individual’s ability to concentrate, work efficiently, and perform daily activities (1). Symptoms of asthenopia, such as dryness, itching, and irritation of the eyes, can be severe enough to hinder personal activities and may even contribute to the development of other eye diseases (2). An effective treatment for asthenopia is to limit the duration of eye use, especially for tasks requiring close-range vision. However, this approach is increasingly challenging in modern society, where digital devices are widely used (3–6). Therefore, identifying risk factors for asthenopia is crucial for improving visual well-being and reducing the risk of experiencing asthenopia.

Meteorological factors such as relative humidity, temperature, and precipitation are environmental factors frequently reported to significantly influence the prevalence of eye diseases. Lv et al. (7) found that the average relative humidity over the past year was negatively associated with cataract likelihood (OR: 0.99; 95% CI: 0.98–0.99), while temperature over the past year was positively associated with cataract likelihood (OR: 1.04; 95% CI: 1.03–1.05) in older adults. In a retrospective analysis using data from the United States National Veterans Administration database, high humidity was found to reduce the risk of dry eye (IRR: 0.927; 95% CI: 0.926–0.927) (8). Our recent work (9) also demonstrated that, compared to arid and semi-arid areas, the average rainfall level in the past year was negatively associated with the likelihood of asthenopia in adults (semi-moist area, OR: 0.70, 95% CI: 0.57–0.86; moist area, OR: 0.77, 95% CI: 0.62–0.95). However, this association was not observed after multivariate analysis.

However, the studies mentioned above analyzed the effects of temperature or relative humidity separately after adjusting for confounding factors, without considering their combined effects. Humidex, developed by Canadian meteorologists Richardson and Masterton (10), is widely used to assess climate comfort as it incorporates both humidity and temperature, which are essential components. Humidex has been shown to better represent the actual perceived ambient temperature (11) and has been used to investigate its comprehensive effects on conditions such as allergic conjunctivitis (12), cardiovascular mortality (11), and bacillary dysentery (13). However, the relationship between humidex and asthenopia remains poorly understood. This study aimed to quantify the associations of humidex (a combined measure of humidity and temperature) with the likelihood and severity of asthenopia among a nationally representative sample of adults in China, utilizing a reliable and validated survey tool.

## 2 Methods

### 2.1 Study population

This cross-sectional nationwide online survey was conducted between June 2020 and March 2022. Invitations were sent to 48,000 Chinese individuals aged 18 and above across 31 provinces in China using the online survey software WJX (Changsha, China) through quota (124 to 4,311 individuals in each province according to the China National Bureau of Statistics percentage of the resident population in 2019) and simple sampling methods.

Participants were provided with information about the survey’s purpose, significance, content, and privacy protection before participating. The Institutional Ethics Committee of the Eye Hospital of Wenzhou Medical University approved the study (No. KYK-2016-8). A total of 34,303 adults who completed the questionnaire were included in the final analysis, yielding a response rate of 71.5%.

### 2.2 Assessment in residential relative humidity and temperature

City-level daily meteorological data on air relative humidity (%) and temperature (°C) for 2020–2022 were obtained from the China Meteorological Data Network.^1^ Daily data were averaged over two-week, monthly, or annual periods to assess residential exposure to relative humidity and temperature for each participant, corresponding to their city of residence. The humidity and temperature exposures in this study were defined as follows: (1) Average relative humidity or temperature over the past 2 weeks, which aligned with the assessment period for asthenopia; (2) Average relative humidity or temperature over the past month; and (3) Average relative humidity or temperature over the past year.

### 2.3 Calculation of humidex

In this study, humidex was used as the exposure indicator, and it was calculated using the following formula (10, 13):


$$\mathrm{Humidex}=Tem+\frac{5}{9}\left\{6.112\;\times \;10\left(\frac{7.5\;\times \;Tem}{237.7+Tem}\right)\times \frac{\mathrm{Hum}}{100}-10\right\}\text{,}$$

where *Tem* and *Hum* indicate the daily average temperature (°C) and relative humidity (%).

Although humidex is dimensionless, it can be interpreted as a dry temperature in °C. For example, if the actual temperature is 30°C but the calculated humidex is 40°C, the perceived temperature would feel like 40°C. The high humidex environment refers to hot/humid conditions, the low humidex environment refers to cold/dry conditions. Humidex was categorized into four levels: Low (<10°C), Normal (10–29.99°C), High (30–39.99°C), and Very High (≥40°C), following previous studies (12, 14, 15).

### 2.4 The definition of asthenopia

The 17-item Asthenopia Survey Questionnaire (ASQ-17) (16, 17), based on Rasch measurement theory and recognized as a reliable and validated tool for assessing asthenopia in the Chinese population, was used in this study. Participants reported the presence of 17 symptoms, including ocular, visual, and systemic, that they had experienced over the past 2 weeks. Each item was scored on a four-point scale: “none” (0), “mild” (1), “moderate” (2), and “severe” (3). The total score was calculated by summing all item scores. Participants with a total score of 13 or higher were classified as having asthenopia. Asthenopia severity was further categorized into three levels based on the ASQ score: Mild (13–17), Moderate (18–33), and Severe (34–51).

### 2.5 Covariates

To minimize the impact of potential confounders, we reviewed literature from the past 10 years in PubMed to identify relevant covariates, focusing on common predictors of asthenopia (3, 4, 9, 18, 19). These variables included age, gender (male or female), season of investigation (warm or cold), geographic region (eastern provinces or central and western provinces), presbyopia status (with or without), history of eye surgery (with or without), average daily duration of near vision activities (e.g., using digital devices, reading) over the past 2 weeks (≤2, 2.01–4, 4.01–6, 6.01–8, or > 8 h per day), daily sleep duration over the past 2 weeks (≤4, 4.01–6, 6.01–8, or > 8 h per day), sleep quality over the past 2 weeks (using a 5-point Likert scale: very good, good, moderate, bad, or very bad), and frequency of heightened anxiety or depression (using a 5-point Likert scale: none, rarely, sometimes, most times, or always). The warm season was defined as April to September, and the cold season from October to March of the following year (12).

### 2.6 Data quality control

Data reproducibility, integrity, and dependability were ensured as part of quality control. The survey allowed only one response per IP address, and all questions had to be completed before submission. Age was calculated using the formula: (response date − birth date) / 365.25. Meteorological data had no omissions.

### 2.7 Statistical analysis

Statistical analyses were conducted using SPSS 25.0 software (SPSS Inc., Chicago, IL). Continuous variables were presented as mean ± standard deviation (SD), while categorical variables were expressed as counts and percentages.

This study explored the effect of humidex on the likelihood and severity of asthenopia. Associations between potential factors and the likelihood of asthenopia were assessed using the independent *t*-test, chi-square test, or Bonferroni test. Factors with a *p*-value below 0.05 were included in a generalized estimation equation (GEE) with an exchangeable correlation structure to adjust for potential confounders. GEE employs a working correlation matrix to evaluate the association structure between observations. This approach not only effectively reduces systematic bias introduced by confounding factors but also significantly enhances the robustness and reliability of parameter estimation (20). In addition, GEE uses a “quasi-likelihood” method which provides efficient and unbiased regression parameters when covariates include both continuous (e.g., age and humidex in this study) and categorical (e.g., gender and humidex grades in this study) variables, regardless of the distribution of the dependent variable (21). Finally, GEE estimates the marginal effect, which is the overall average effect after controlling for confounding factors, rather than the individual-level effect (22). We used an ordinal logit link function with “Non-asthenopia” as the reference group and reported. Odds Ratios (OR) and 95% confidence intervals (CIs). A *p*-value of less than 0.05 was considered statistically significant.

## 3 Results

### 3.1 Asthenopia and demographic factors

A total of 34,303 participants were included in this study. The overall prevalence of asthenopia was 38.8%, with 17.1% classified as mild, 18.5% as moderate, and 3.2% as severe. Participants’ ages ranged from 18.62 to 101.71 years, with a mean age of 32.76 ± 10.68 years. As shown in Table 1, the distribution of gender and geographic region was relatively balanced (45.9% male, 57.9% from eastern China). The majority (70.4%) were surveyed during the warm season. About 15.4% of participants had presbyopia, and 11.2% had a history of eye surgery. Nearly a quarter (23.9%) engaged in near vision activities for 4.01 to 6 h per day, and over half (61.2%) reported sleeping between 6.01 to 8 h per day. Sleep quality was rated as moderate by 41.0% of participants, and 40.7% reported sometimes experiencing heightened anxiety or depression.

**Table 1:** Demographics of the study participants.

	All (*n* = 34,303)	Non-asthenopia (*n* = 20,993)	Asthenopia (*n* = 13,310)	Z/t/𝜒^2^ value*	*p*-value
Having asthenopia, *n* (%)	13,310 (38.8)				
Asthenopia grades, *n* (%)					
Mild: ASQ score13-17			5,880 (17.1)		
Moderate: ASQ score 18–33			6,335 (18.5)		
Severe: ASQ score 34–51			1,095 (3.2)		
Average relative humidity of the past 2 weeks, %, mean ± SD (range)	73.18 ± 11.80 (23.95, 96.39)	73.58 ± 11.63 (26.42, 96.39)	72.56 ± 12.02 (23.95, 93.91)	7.840^a^	**<0.001**
Average temperature of the past 2 weeks, °C, mean ± SD (range)	19.72 ± 9.31 (−23.06, 30.90)	19.44 ± 9.41 (−23.06, 30.90)	20.15 ± 9.24 (−22.33, 30.90)	−6.826^a^	**<0.001**
Average humidex of the past 2 weeks, °C, mean ± SD (range)	25.07 ± 14.26 (−28.25, 45.75)	24.59 ± 14.35 (−28.25, 45.75)	25.83 ± 14.18 (−27.53, 45.29)	−7.808^a^	**<0.001**
Humidex grades, *n* (%)				117.140^b^	**<0.001**
Low: <10°C	5,741 (16.7)	3,611 (17.2)	2,130 (16.0)		**0.004**^**c**^
Normal: 10–29.99°C	12,203 (34.6)	7,851 (37.4)	4,352 (32.7)		**<0.001**^**c**^
High: 30–39.99°C	11,460 (34.4)	6,655 (31.7)	4,805 (36.1)		**<0.001**^**c**^
Very high: ≥ 40°C	4,899 (14.3)	2,876 (13.7)	2023 (15.2)		**0.003** ^**c**^
Average humidex of the past 1 month, °C, mean ± SD (range)	24.58 ± 14.37 (−25.69, 43.05)	24.13 ± 14.33 (−25.69, 43.05)	25.29 ± 14.37 (−25.69, 43.05)	−7.268^b^	**<0.001**
Average humidex of the past 1 year, °C, mean ± SD (range)	15.88 ± 8.52 (−2.55, 34.36)	15.79 ± 8.64 (−2.55, 34.36)	16.03 ± 8.32 (−2.55, 34.36)	−2.550^b^	**<0.001**
Covariates					
Age, years, mean ± SD (range)	32.76 ± 10.68 (18.62, 101.71)	32.31 ± 10.22 (18.72, 99.58)	33.46 ± 11.35 (18.62,101.71)	−9.754^a^	**<0.001**
Male, *n* (%)	15,755 (45.9)	9,965 (47.5)	5,790 (43.5)	51.619^b^	**<0.001**
East China, *n* (%)	19,859 (57.9)	12,127 (57.8)	7,732 (58.1)	0.352^b^	0.553
Warm season, *n* (%)	24,151 (70.4)	14,695 (70.0)	9,756 (73.3)	43.974^b^	**<0.001**
With presbyopia status, *n* (%)	5,284 (15.4)	2,761 (13.2)	2,523 (19.0)	210.545^b^	**<0.001**
With a history of eye surgery, *n* (%)	3,850 (11.2)	2,440 (11.6)	1,410 (10.6)	8.663^b^	**0.003**
Duration of near vision activity, *n* (%)				208.856^b^	**<0.001**
≤2 h per day	3,016 (8.8)	2071 (9.9)	945 (7.1)		**<0.001**^**c**^
2.01–4 h per day	6,755 (19.7)	4,336 (20.7)	2,419 (18.2)		**<0.001**^**c**^
4.01–6 h per day	8,183 (23.9)	5,090 (24.2)	3,093 (23.3)		**0.001** ^**c**^
6.01–8 h per day	7,469 (21.7)	4,540 (21.6)	2,929 (22.0)		0.683^c^
>8 h per day	8,800 (25.9)	4,956 (23.6)	3,924 (29.5)		**<0.001** ^**c**^
Sleep duration, *n*(%)				629.097^b^	**<0.001**
>8 h per day	4,902 (14.3)	3,375 (16.1)	1,527 (11.5)		**<0.001**^**c**^
6.01–8 h per day	20,993 (61.2)	13,407 (63.9)	7,586 (57.0)		**<0.001** ^**c**^
4.01–6 h per day	6,067 (17.7)	2,982 (14.2)	3,085 (23.2)		**<0.001** ^**c**^
≤4 h per day	2,341 (6.8)	1,229 (5.8)	1,112 (8.3)		**<0.001** ^**c**^
Sleep quality, *n* (%)				2360.616^b^	**<0.001**
Very good	6,847 (20.0)	5,397 (25.7)	1,450 (10.9)		**<0.001** ^**c**^
Good	10,137 (29.6)	6,818 (32.5)	3,319 (24.9)		**<0.001** ^**c**^
Moderate	14,084 (41.0)	7,679 (36.6)	6,405 (48.1)		**<0.001** ^**c**^
Bad	2,764 (8.0)	996 (4.7)	1768 (13.3)		**<0.001** ^**c**^
Very bad	471 (1.4)	103 (0.5)	368 (2.8)		**<0.001** ^**c**^
Frequency of heightened anxiety or depression, *n* (%)				3890.745^b^	**<0.001**
None	8,676 (25.2)	7,249 (34.5)	1,427 (10.8)		**<0.001** ^**c**^
Rarely	7,982 (23.3)	5,294 (25.2)	2,688 (20.2)		**<0.001** ^**c**^
Sometimes	13,953 (40.7)	7,347 (35.0)	6,606 (49.6)		**<0.001** ^**c**^
Always	3,004 (8.8)	952 (4.5)	2052 (15.4)		**<0.001** ^**c**^
Most times	688 (2.0)	151 (0.8)	537 (4.0)		**<0.001** ^**c**^

* Comparison of demographic information of participants with asthenopia and non-asthenopia. OR with statistically significant were listed in bold.

a Independent *T*-test.

b Chi-square test.

c Bonferroni test.

Asthenopia prevalence was significantly higher among participants who were older, female, surveyed during the warm season, had presbyopia, and lacked a history of eye surgery. A higher prevalence was also associated with engaging in more than 8 h of daily near vision activity, slept 4 h or less per day, had poorer sleep quality (moderate, bad, or very bad), and more frequently (sometimes, most times, or always) experienced heightened anxiety or depression. No significant differences in prevalence were observed based on geographic region, as indicated in Table 1.

### 3.2 Humidex exposure

The average humidex (mean ± SD) for the past 2 weeks, 1 month, and 1 year were 25.07 ± 14.26°C, 24.58 ± 14.37°C, and 15.88 ± 8.52°C, respectively. These three humidex values were significantly higher in participants with asthenopia compared to those without (25.83 ± 14.18°C vs. 24.59 ± 14.35°C, 25.29 ± 14.37°C vs. 24.13 ± 14.33°C, and 16.03 ± 8.32°C vs. 15.79 ± 8.64°C, respectively, all *p*-values <0.001). Humidity over the past 2 weeks ranged from 23.95 to 96.39%, and temperatures ranged from −23.06°C to 30.90°C. The average relative humidity and temperature data are also summarized in Table 1.

### 3.3 Associations of humidex with the likelihood and severity of asthenopia

The average humidex over the past 2 weeks (ranging from −28.25 to 45.75°C) was positively correlated with the likelihood and severity of asthenopia compared to non-asthenopia. Each 1°C increase in the average humidex over this period was associated with a 0.5% rise in asthenopia prevalence (OR: 1.005, 95% CI: 1.003–1.006, *p*-value <0.001). Similarly, each 1°C increase was linked to a 0.4% rise in the severity grade of asthenopia (OR: 1.004, 95% CI: 1.003–1.006, *p*-value <0.001), as shown in Table 2. Subgroup analyses indicated that these associations were consistent across various risk factors for asthenopia, including gender, season, presbyopia status, duration of near vision activities, sleep duration, sleep quality, and frequency of heightened anxiety or depression. The effects of humidex were more pronounced among females, those with presbyopia, those engaged in more than 8 h of daily near vision activity, those with a daily sleep duration of 4.01–6 h, those reporting poor sleep quality, and those who always experienced heightened anxiety or depression. However, average humidex showed no significant effects for participants surveyed in the cold season, those with or without a history of eye surgery, and those with extreme levels of certain risk factors (e.g., daily near vision activity duration of ≤2 h, daily sleep duration of ≤4 h, very poor sleep quality, and frequent anxiety or depression; see Table 3).

**Table 2:** Effects of air relative humidex on asthenopia among Chinese adults during 2020–2022: odds ratios (OR) from GEE models (*n* = 34,303).

	Having asthenopia^a^	*p*-value	Asthenopia grades^a^	*p*-value
	OR (95% CI)		OR (95% CI)	
Average humidex of the past 2 weeks, °C	1.005 (1.003–1.006)	**<0.001**	1.004 (1.003–1.006)	**<0.001**
Covariates				
Age, years	1.004 (1.002–1.006)	**0.001**	1.003 (1.002–1.006)	**<0.001**
Male (female*)	0.950 (0.923–0.978)	**<0.001**	0.978 (0.961–0.995)	**0.010**
Warm season (cold season*)	1.020 (0.971–1.072)	0.422	1.029 (0.997–1.061)	0.072
Presbyopia status (without*)	1.552 (1.461–1.649)	**<0.001**	1.301 (1.255–1.350)	**<0.001**
History of eye surgery (without*)	1.015 (0.970–1.062)	0.516	1.021 (0.994–1.048)	0.138
Duration of near vision activity (≤2 h per day*)				
2.01–4 h per day	1.055 (0.992–1.120)	0.086	1.015 (0.982–1.050)	0.369
4.01–6 h per day	1.099 (1.035–1.168)	**0.002**	1.037 (1.003–1.073)	**0.033**
6.01–8 h per day	1.155 (1.085–1.229)	**<0.001**	1.067 (1.031–1.105)	**<0.001**
>8 h per day	1.281 (1.204–1.362)	**<0.001**	1.141 (1.102–1.181)	**<0.001**
Sleep duration (> 8 h per day*)				
6.01–8 h per day	0.993 (0.950–1.037)	0.747	0.980 (0.956–1.004)	0.106
4.01–6 h per day	1.252 (1.187–1.321)	**<0.001**	1.141 (1.105–1.179)	**<0.001**
≤4 h per day 4	1.656 (1.545–1.775)	**<0.001**	1.328 (1.274–1.384)	**<0.001**
Sleep quality (very good*)				
Good	1.222 (1.168–1.278)	**<0.001**	1.093 (1.069–1.117)	**<0.001**
Moderate	1.450 (1.388–1.514)	**<0.001**	1.220 (1.192–1.249)	**<0.001**
Bad	1.874 (1.757–1.998)	**<0.001**	1.579 (1.512–1.648)	**<0.001**
Very bad	2.327 (2.013–2.690)	**<0.001**	2.110 (1.912–2.330)	**<0.001**
Frequency of heightened anxiety or depression (None*)				
Rarely	1.649 (1.579–1.723)	**<0.001**	1.244 (1.218–1.271)	**<0.001**
Sometimes	2.195 (2.107–2.288)	**<0.001**	1.537 (1.505–1.570)	**<0.001**
Always	3.421 (3.219–3.635)	**<0.001**	2.336 (2.244–2.433)	**<0.001**
Most times	4.245 (3.776–4.773)	**<0.001**	3.010 (2.773–3.268)	**<0.001**

CI, confidence interval; GEE, generalized estimation equation. Reference group.

a Used non-asthenopia group as reference; OR with statistically significant were listed in bold.

**Table 3:** Effects of air relative humidex on asthenopia: odds ratios (OR) from hierarchical GEE models (*n* = 34,303).

Group	Having asthenopia^a^	*p*-value	Asthenopia grades^a^	*p*-value
	OR (95% CI)		OR (95% CI)	
Total	1.005 (1.003–1.006)	**<0.001**	1.004 (1.003–1.006)	**<0.001**
Gender				
Male	1.003 (1.001–1.006)	**<0.001**	1.004 (1.002–1.006)	**0.001**
Female	1.009 (1.005–1.012)	**<0.001**	1.005 (1.003–1.007)	**<0.001**
Investigated season				
Warm	1.018 (1.014–1.021)	**<0.001**	1.019 (1.015–1.022)	**<0.001**
Cold	1.002 (0.999–1.006)	0.178	1.001 (0.998–1.005)	0.473
Presbyopia status				
With	1.009 (1.003–1.015)	**0.003**	1.011 (1.005–1.016)	**<0.001**
Without	1.007 (1.005–1.010)	**<0.001**	1.009 (1.006–1.011)	**<0.001**
History of eye surgery				
With	1.004 (0.999–1.009)	0.102	1.004 (1.000–1.009)	0.061
Without	0.997 (0.993–1.000)	0.086	0.998 (0.996–1.001)	0.201
Duration of near vision activity				
≤2 h per day	1.003 (0.997–1.010)	0.315	1.004 (0.997–1.010)	0.257
2.01–4 h per day	1.007 (1.003–1.011)	**<0.001**	1.007 (1.003–1.011)	**<0.001**
4.01–6 h per day	1.005 (1.002–1.008)	**0.004**	1.007 (1.004–1.010)	**<0.001**
6.01–8 h per day	1.006 (1.003–1.010)	**<0.001**	1.008 (1.004–1.011)	**<0.001**
>8 h per day	1.013 (1.006–1.020)	**<0.001**	1.009 (1.006–1.012)	**<0.001**
Sleep duration				
>8 h per day	1.008 (1.003–1.012)	**0.001**	1.008 (1.003–1.012)	**0.001**
6.01–8 h per day	1.006 (1.004–1.009)	**<0.001**	1.007 (1.005–1.009)	**<0.001**
4.01–6 h per day	1.009 (1.005–1.013)	**<0.001**	1.008 (1.005–1.012)	**<0.001**
≤4 h per day 4	0.996 (0.988–1.004)	0.292	0.996 (0.990–1.003)	0.325
Sleep quality				
Very good	1.008 (1.004–1.012)	**<0.001**	1.008 (1.004–1.012)	**<0.001**
Good	1.005 (1.002–1.008)	**<0.001**	1.006 (1.003–1.009)	**<0.001**
Moderate	1.007 (1.004–1.009)	**<0.001**	1.007 (1.005–1.009)	**<0.001**
Bad	1.010 (1.004–1.016)	**0.001**	1.011 (1.006–1.016)	**<0.001**
Very bad	0.998 (0.982–1.014)	0.764	0.997 (0.985–1.010)	0.997
Frequency of heightened anxiety or depression				
None	1.006 (1.001–1.012)	**0.033**	1.009 (1.005–1.014)	**<0.001**
Rarely	1.003 (1.000–1.007)	**0.011**	1.004 (1.000–1.007)	**0.038**
Sometimes	1.007 (1.004–1.009)	**<0.001**	1.007 (1.005–1.009)	**<0.001**
Always	1.011 (1.007–1.015)	**<0.001**	1.012 (1.008–1.015)	**<0.001**
Most times	1.003 (0.999–1.006)	0.136	1.008 (0.997–1.018)	0.144

CI, confidence interval; GEE, generalized estimation equation.

a Used non-asthenopia group as reference; OR with statistically significant were listed in bold.

Sensitivity analyses were conducted to assess the robustness of our findings by examining the average humidex over the past 2 weeks (Model I), the past month (Model II), and the past year (Model III). The results were consistent with the main findings from Model I, as presented in Table 4.

**Table 4:** Effects of air relative humidex on asthenopia: odds ratios (OR) from GEE models (*n* = 34,303).

	Having asthenopia^a^	*p*-value	Asthenopia grades^a^	*p*-value
	OR (95% CI)		OR (95% CI)	
Model 1				
Average humidex of the past 2 weeks, °C	1.005 (1.003–1.006)	**<0.001**	1.004 (1.003–1.006)	**<0.001**
Model 2				
Average humidex of the past 1 month, °C	1.004 (1.003–1.005)	**<0.001**	1.003 (1.002–1.004)	**<0.001**
Model 3				
Average humidex of the past of the past 1 year, °C	1.002 (1.001–1.004)	**0.008**	1.002 (1.001–1.003)	**<0.001**

Covariates are the same as in Table 2, the odds ratios of which are not listed. CI, confidence interval; GEE, generalized estimation equation.

a Used non-asthenopia group as reference; OR with statistically significant were listed in bold.

**Figure 1:**
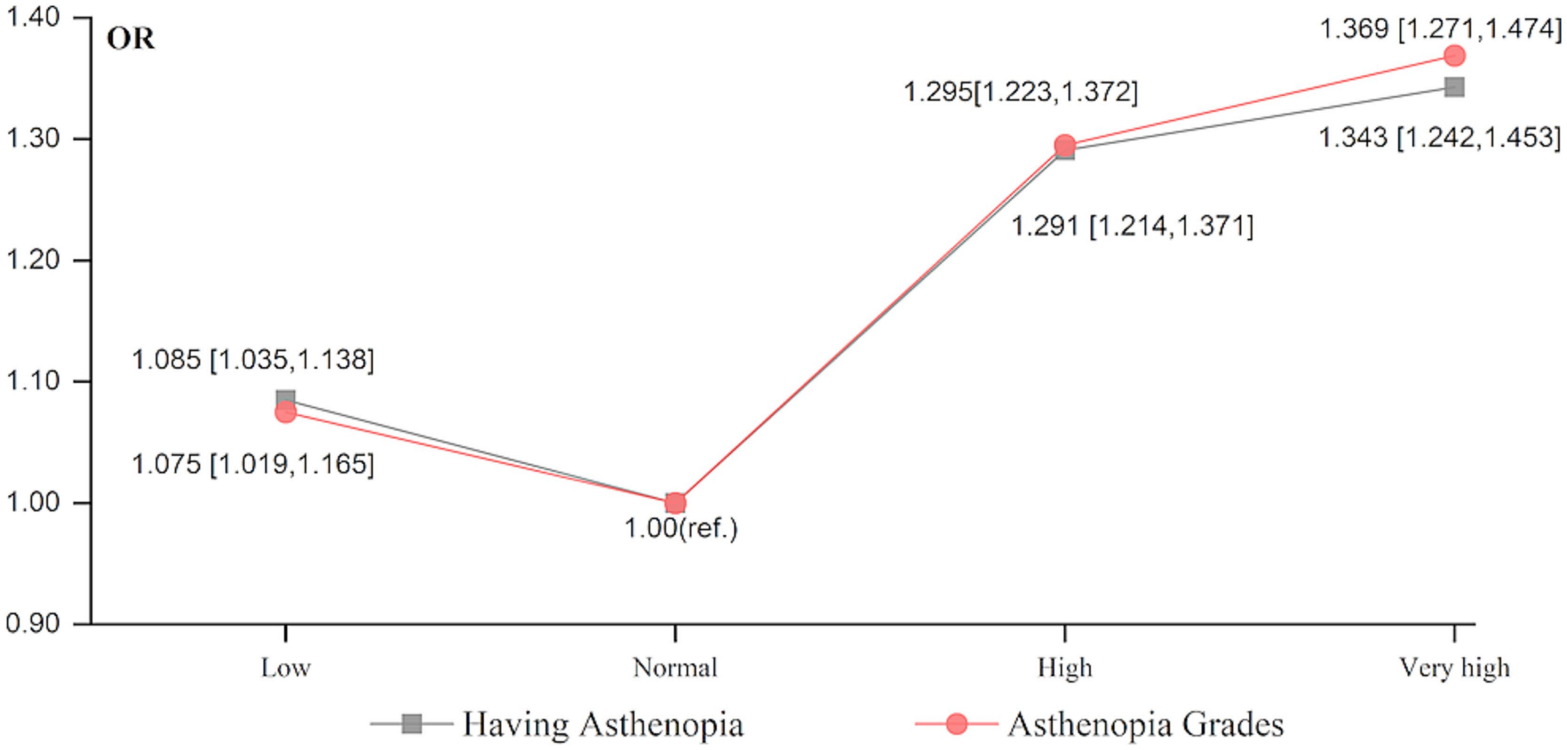
Effects of categorical humidex on having asthenopia and asthenopia grades. Covariates are the same as in Table 2.

Additionally, to explore the potential nonlinear relationship between humidex and asthenopia, humidex was modeled as a categorical variable with “normal (10–29.99°C)” as the reference group. According to Figure 1, a nonlinear J-shaped relationship was observed between the average humidex over the past 2 weeks and the likelihood and severity of asthenopia. Humidex generally showed a positive association with the likelihood and severity of asthenopia within the “normal (10–29.99°C), high (30–39.99°C), very high (≥ 40°C)” range, there was a noticeable negative trend when the humidex was within the “low (<10°C), normal (10–29.99°C)” range. Moreover, the OR values of high (1.291 and 1.295 respectively) and very high (1.343 and 1.369 respectively) humidex were higher than low (1.085 and 1.075 respectively) humidex. These indicate that both high and low humidex levels increased the risk of asthenopia when deviating from the optimal humidex range of 10–29.99°C, the adverse effect of high humidex (≥ 30°C) on asthenopia was more pronounced than that of low humidex (<10°C).

## 4 Discussion

The prevalence of asthenopia is particularly concerning as it is increasingly becoming one of the most common ocular conditions among individuals (9, 18, 19). To our knowledge, this is the first study to examine the impact of humidex on the likelihood and severity of asthenopia in adults, including potential variations across different subgroups. Our findings indicate that both high (≥ 30°C) and low (< 10°C) humidex exposure are associated with an increase the risk of asthenopia and its severity in adults, even after adjusting for demographic factors and lifestyle patterns, including age, gender, season, geographic region, mental status, medical history, and lifestyle. The associations between humidex and asthenopia were stronger among females, those with presbyopia, those engaging in more than 8 h of daily near vision activity, those with a daily sleep duration of 4.01–6 h, those with poor sleep quality, and those who consistently experienced heightened anxiety or depression. Overall, our study provides a comprehensive understanding of humidex’s impact on asthenopia in adults and offers important insights for future public health interventions aimed at reducing weather-related health risks.

We found that each 1°C increase in the average humidex over the past 2 weeks (range − 28.25 to 45.75°C) was associated with a 0.5% increase in asthenopia prevalence (OR: 1.005, 95% CI: 1.003–1.006, *p*-value <0.001) and a 0.4% increase in asthenopia severity (OR: 1.004, 95% CI: 1.003–1.006, *p*-value <0.001). Additionally, a nonlinear J-shaped relationship was observed, indicating that both low and high humidex levels increased the risk of asthenopia when deviating from the optimal humidex range of 10–29.99°C.

Although limited epidemiological studies have specifically examined the health effects of humidex on asthenopia, similar findings have been reported in studies investigating the effects of humidity or temperature on symptoms related to asthenopia, such as eye dryness, ocular discomfort, and eye soreness. A study involving 16,824 individuals in South Korea (23) found that a 5% increase in humidity levels (ranging from 50.0 to 80.0%) was associated with a reduction in symptoms and diagnoses of dry eye disease (symptoms: OR, 0.87; 95% CI, 0.77–0.98; diagnosis: OR, 0.86; 95% CI, 0.76–0.97). Conversely, as relative humidity decreases, the rate of tear evaporation increases, worsening dry eye symptoms (24). However, not all studies have shown an inverse relationship between humidity and asthenopia-related symptoms. A U.S. study (25) involving 97 individuals found a positive association between humidity (ranging from 35.0 to 96.0%) and the Ocular Surface Disease Index (R: 0.30, 95% CI: 0.07–0.49, *p* = 0.01), as well as between humidity and inflammation (R: 0.32, 95% CI: 0.10–0.51, *p* = 0.01). This was attributed to the interaction between humidity and particulate matter, rather than a direct effect of humidity on ocular discomfort. High humidity can prolong the airborne duration of particulate matter and increase its mass and size (hygroscopic effect) (26). Additionally, elevated humidity can promote the production and aerosolization of bioaerosols, such as mold spores and endotoxins, which have been associated with ocular surface inflammation (27). This suggests the existence of a “Goldilocks” zone for humidity, where both low and high levels can have negative effects, likely due to interactions with particulate matter. The strongest evidence comes from randomized controlled trials conducted in environmental chambers, which demonstrate that eye dryness and ocular discomfort can be improved by adjusting humidity (28) and temperature (29).

Similarly, both extreme outdoor and indoor temperatures are associated with asthenopia-related symptoms. An experimental study (30) found that temperatures above 40°C can alter the properties of meibomian gland lipids and disrupt the tear film, potentially worsening dry eye symptoms. In a South Korean population, the diagnosis of dry eye disease was positively associated with outdoor temperature, with a 1°C increase corresponding to an OR of 1.076 (95% CI: 1.009–1.148) (31). A crossover study evaluating a cooler indoor environment (between 22.2°C and 25.6°C) showed that a 1°C decrease in temperature improved symptoms related to asthenopia, such as eye dryness, itching, and irritation, by 19% (95% CI: 8–31%) (32). However, Song et al. (33) found in a study of 33 individuals that tear osmolarity decreases as temperature drops from 18°C to 6°C, contributing to tear film instability, dry eye, and ocular inflammation, ultimately leading to asthenopia. There is a general consensus that extreme humidity and temperature are not ideal for eye health, and it appears that a moderate humidex is optimal—a conclusion supported by the findings of this study.

Other weather conditions such as wind and sunlight appear to increase eye dryness. In an observational survey-based study of 5 European countries (*n* = 738), the environmental factors that most impacted dry eye symptoms were wind, sunlight, and heat, affecting 71%, 60%, and 41% of respondents, respectively (34). Outdoor air pollution has also been linked to eye dryness. In a Chinese retrospective case-crossover study of 5,062 individuals, same day exposure of PM2.5 (OR: 1.02, 95% CI: 1.01–1.03, *p*-value < 0.01) and PM10 (OR: 1.01, 95% CI: 1.003–1.02, *p*-value< 0.01) increased the risk of an outpatient dry eye visit (35). In a Swedish study, 89 children aged 7–18 with known pollen allergy, their ocular symptoms were significantly dependent on pollen count (*p*-value < 0.01) (36). Similarly, exposure to an adverse indoor environment can negatively impact eye dryness. In a U.S. study of 97 individuals, a 1 unit increase in PM2.5 of home environment was associated with a 1.59 increased in Ocular Surface Disease Index score (95% CI: 0.58–2.59, *p* = 0.002) (25). Similar findings have been observed in China. Among 3,485 randomly selected healthy adults, those living in homes with multiple signs of dampness and mold showed an increased risk of ocular itching, irritation, or burning (OR: 3.20, 95% CI: 1.67–6.15, *p*-value < 0.01) (37). To conclude, studies have found associations between outdoor and indoor environmental factors exposure and painful eye dryness, but no direct correlation was found between environmental factors and asthenopia.

Our research focused on the effect of humidex on asthenopia, an index that combines humidity and temperature to reflect perceived temperature. Although the biological mechanisms underlying the influence of humidity and temperature on asthenopia are not fully understood in this study, we speculate that low humidex environments (cold/dry conditions) may induce and aggravate asthenopia by disrupting tear film stability, while high humidex environments (hot/humid conditions) may act through disrupting tear film stability, exacerbating inflammation or particulate effects. This may also be the reason why the adverse effect of high humidex on asthenopia was more pronounced than that of low humidex, as shown in Figure 1. Existing evidence suggests that both humidity and temperature have non-linear relationships with health and that their combined effect may significantly exceed the sum of their individual effects (38). An experimental study (39) demonstrated that the human body’s responses to cold and heat—such as sensation, acceptability, and comfort—change markedly as relative humidity increases. Physiological data indicate that in hot environments, the body regulates its core temperature primarily through perspiration (40). However, high humidity can hinder the cooling process, especially in warm, humid climates, by reducing heat loss through evaporation. When body temperature becomes too high, it may lead to autonomic nervous system dysfunction and a reduction in secretion, ultimately impairing the tear film and contributing to asthenopia (41). These results showed the necessity of taking timely and protective measures to prevent people from being exposed to both high (≥ 30°C, hot/humid conditions) and low (<10°C, cold/dry conditions) humidex, especially high humidex, to effectively reduce asthenopia.

This study has several strengths. Firstly, it is the first study to apply humidex as a metric to investigate its association with asthenopia. We used humidex as a potential indicator because it combines ambient temperature and relative humidity, potentially exerting a more direct and pronounced impact on human health than either factor alone. Secondly, we thoroughly analyzed the effects of different humidex levels, providing important insights into targeted preventive measures. Thirdly, our analytical model controlled for the confounding effects of other variables, including age, gender, mental status, medical history, and lifestyle, ensuring the robustness of our results.

Nevertheless, our study has several limitations. Firstly, our exposure assessment is limited as it does not account for individual differences in exposure. The temperature and humidity data used to calculate the humidex were collected from fixed-site outdoor monitors and reflected the overall exposure levels of residents in a given area. Although this method is commonly used in environmental epidemiology studies, it fails to account for adaptive behaviors such as air conditioning use, reduced outdoor exposure, or local preventive and intervention measures, potentially leading to misclassification. Secondly, we relied on a self-reported questionnaire to assess asthenopia instead of using objective measurements. The targeting indicator (mean person location) of ASQ-17 was −0.80 logit (which met the standard of less than 1.0 logit) (16). The negative value indicates that the questionnaire was generally challenging for the study population, potentially excluding cases of mild asthenopia. This would strengthen the associations between variables, but it might also directly exclude uncertain risk factors (such as the history of eye surgery in this study), missing influencing factors. Nevertheless, our study benefited from a large sample size, which aimed to minimize bias, and relied on the reliable and validated ASQ-17 survey tool. Additionally, we assessed participants’ subjective symptoms over the preceding 2 weeks to reduce recall bias. Furthermore, as a cross-sectional study, we indeed could only identify a concurrent and causal relationship between humidex and asthenopia. Further researches from controlled environmental chambers and perspective of molecular biology are needed to investigate the causal relationship between humidex and asthenopia development.

## 5 Conclusion

Climate change presents new challenges to public health, and vulnerable populations should be prioritized in asthenopia prevention and interventions. These populations include women, individuals with presbyopia, those engaging in more than 8 h of daily near vision activity, those with daily sleep duration of 4.01–6 h, those with poor sleep quality, and those who consistently experience heightened anxiety or depression. The optimal humidex range for minimizing asthenopia risk is identified as 10–29.99°C, while both high (≥30°C) and low (<10°C) humidex conditions, particularly the former, require enhanced protection for vulnerable populations. These findings provide guidance for relevant authorities to implement targeted preventive measures and optimize medical resource allocation. Further research is needed to explore the role of humidex as an influencing factor.

## Data Availability

The raw data supporting the conclusions of this article will be made available by the authors, without undue reservation.

## Footnotes

^1^ http://data.cma.cn/
